# Evolution of a Yeast With Industrial Background Under Winemaking Conditions Leads to Diploidization and Chromosomal Copy Number Variation

**DOI:** 10.3389/fmicb.2018.01816

**Published:** 2018-08-03

**Authors:** Ana Mangado, Pilar Morales, Ramon Gonzalez, Jordi Tronchoni

**Affiliations:** Instituto de Ciencias de la Vid y del Vino, Gobierno de La Rioja, Consejo Superior de Investigaciones Científicas, Universidad de La Rioja, Logroño, Spain

**Keywords:** evolution, adaptive laboratory evolution, aneuploidies, wine fermentation, MAT locus switching

## Abstract

Industrial wine yeast strains show genome particularities, with strains showing polyploid genomes or chromosome copy number variations, being easier to identify. Although these genomic structures have classically been considered transitory steps in the genomic adaptation to new environmental conditions, they may be more stable than thought. These yeasts are highly specialized strains able to cope with the different stresses associated with the fermentation process, from the high osmolarity to the final ethanol content. In this work, we use adaptive laboratory evolution, focusing on the initial steps of the fermentation process, where growth rate is maximum, to provide new insights into the role of the different genomic and chromosomic rearrangements that occur during adaptation to wine conditions, and providing an understanding of the chronology of the different evolutionary steps.

## Introduction

Industrial yeast strains are an essential element in the production of various goods for human use, from the traditional food related, bread, beer, and wine to the more recently and technological, bioethanol, heterologous proteins, and low-molecular-weight compounds (Pretorius, 2000; Mattanovich et al., 2014; de Vries et al., 2017). Together, the traditional yeast strains, and those that have emerged more recently to enable new industrial applications, have a number of genomic characteristics in common that distinguish them from wild strains. At genomic level, domesticated yeast strains show higher frequencies of polyploid genomes compared to strains found in nature, this is even more common in industrial hybrid strains, while at chromosomal level it is easy to find variations in the number of copies of chromosomes (chromosome copy number variation, CCNV) (Sipiczki, 2011; Steensels et al., 2014; de Vries et al., 2017; Guillamón and Barrio, 2017).

Diploidization and aneuploidies have been described as evolutionary phenomena that allow adaptation to a new niche where growth conditions are different or particular. Being particularity beneficial under unstable circumstances (Comai, 2005; Sheltzer and Amon, 2011). These types of adaptations have been considered generally transitory with the main role being to generate the appropriate conditions to allow gene speciation, by increasing the number of copies of genes or allowing the presence of SNPs in heterozygosis. Access to next-generation sequencing (NGS) technologies has shown that this may not be entirely true and that such variations in the number of chromosomes may be more stable than originally thought (Mulla et al., 2014; de Vries et al., 2017). Recently, the sequencing of some traditional wine and beer yeast strains has shown that these strains had aneuploidies in their genome, and at least under biotechnology conditions they are stable, although in lab conditions they can lose these aneuploidies (Borneman et al., 2011; van den Broek et al., 2015).

An excellent tool for the production of yeast strains with biotechnological applications is the adaptive laboratory evolutions (ALE) technique (Querol et al., 2003). This methodology has succeeded in providing yeast strain with the desired industrial phenotype which, being GMO free, provides a great advantage by allowing its later commercialization. It is also a great tool for observing yeast evolution by controlling phenotypic and genomic changes during hundreds of yeast generations in a relatively short time. This can be achieved by providing continuous culture conditions using a chemostat and controlling the yeast duplication rate, while keeping a steady environment with the desired sugar and metabolite concentrations (Portnoy et al., 2011; Steensels et al., 2014).

Among the oldest domesticated yeast strains are those related with winemaking (Pretorius, 2000). These highly specialized strains have become adapted by evolution to growth in a difficult environment with many stress factors. From the initial high osmolarity due to high sugar concentrations to the high ethanol content derived from the metabolized sugars (Bauer and Pretorius, 2000). Although in natural fermentations we can find many different yeast species, the domesticated *Saccharomyces cerevisiae* is able to outcompete other species early in the fermentation stages thanks to its high sugar processing rates and its capacity to produce ethanol, coupled with strong resistance to the toxicity of this metabolite (Jolly et al., 2014). Therefore, the initial stages of fermentation are crucial for *S. cerevisiae* to prevail over other yeast species.

This work tries to provide a deeper understanding of the genomic changes that need to have occurred in *S. cerevisiae* for it to become highly specialized in winemaking, focusing on the early stages of the fermentation process. To do so, we have decided to use a different strategy. We took a segregant of the commercial yeast strain EC1118, selected after showing a loss of fermentation performance compared to the original wine strain, and evolved it for more than 250 generations. We recovered three different evolved strains with an improved performance as compared to the initial segregant strain and closer in fermentation capacity to the original strain. The genomic analysis of these strains sheds new light in understanding what the chronological processes are that allowed this to happen.

## Materials and Methods

### Yeast Strains

The yeast strain subjected to the evolution experiment is a segregant strain of the commercial yeast strain *S. cerevisiae* EC1118 (Lallemand, Canada) *MAT*a/α; *HO*/*ho* (heterothallic). This strain, named N2 (*MAT*α, *ho*), was obtained by microdissection of EC1118 ascospores. The evolved strains selected after the evolution of N2 are, NV3, NV6, and NV15.

### Media and Cultivation Conditions

Pre-cultures were grown in a YPD (1% yeast extract, 2% peptone, and 2% glucose) medium for 48 h at 25°C and 150 rpm. Then they were centrifuged at 2,200 × *g*, for 15 min at room temperature, and washed twice with distilled water.

YPD agar plates (20 g/L agar) were used for the general cultivation of yeast strains. YPG (10 g/L yeast extract, 20 g/L peptone, and 30 ml/L glycerol) and YPG agar plates (20 g/L agar) were used as pre-sporulation medium and to evaluate petite phenotypes. Potassium acetate (1%) was prepared as sporulation medium.

For adaptive evolution a simulated synthetic grape must medium was prepared with 25% sugar (v/v), equimolecular concentration of glucose and fructose; 6 g/L DL-malic acid, 6 g/L citric acid, 1.7 g/L YNB (without amino acids and without ammonium sulphate), 306 mg/L NH_4_Cl, 13 mg/L tyrosine, 400 mg/L L-proline, 333 mg/L L-glutamine, 245 mg/L L-arginine, 116 mg/L L-tryptophan, 97 mg/L L-alanine, 80 mg/L L-glutamic acid, 52 mg/L L-serine, 50 mg/L L-threonine, 32 mg/L L-leucine, 29 mg/L L-aspartic acid, 29 mg/L L-valine, 25 mg/L L-phenylalanine, 22 mg/L L-isoleucine, 23 mg/L L-histidine, 21 mg/L L-methionine, 13 mg/L L-tyrosine, 12 mg/L L-glycine, 11 mg/L L-lysine, 14 mg/L L-cysteine, and 83 mg/L L-uridine. All the amino acids with the exception of tyrosine, were added in a 50× solution prepared, sterilized by filtration, aliquoted, and then kept at -20°C until required. Glucose and fructose were autoclaved separately and added to the rest of the medium. Concentrations of anaerobic factors were 15 mg/L ergosterol, 5 mg/L sodium oleate, and 0.5 mL/L tween 80. The pH was adjusted to 3.5 with 10 N KOH.

### Laboratory Adaptive Evolution

Continuous cultures were run in DASGIP bioreactor vessels (DASGIP AG, Jülich, Germany) using a working volume of 300 mL of synthetic must media. The continuous system station was kept in a temperature-controlled chamber (Medilow, JP Selecta, Abrera, Barcelona, Spain) set at 28°C, and homogenization of the culture was achieved by magnetic stirring. CO_2_ release and limited gas exchange ensured near anaerobic growth conditions.

Experimental evolution started with an initial OD_600_ of 0.2 (6.0 log10 CFU/mL). After 24 h of batch culture, peristaltic pumps maintained the continuous culture by feeding the system with synthetic must media with a dilution rate adjusted to 0.20 h^-1^. OD_600_ and dilution rate were checked daily while metabolite content, pH and strain contaminations were monitored to ensure that the system worked properly. Cell samples were taken approximately every 50 generations and stored at -80°C in a 20% glycerol solution. Evolution conditions were maintained for around 270 generations. Once the evolution experiment was finished, 35 colonies were randomly selected for phenotypic characterization.

### Fermentation Kinetics

**FIGURE 1:**
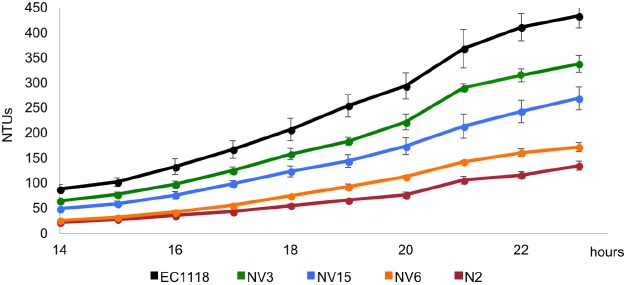
Growth kinetics in synthetic grape must of the original industrial strain EC1118 (*black*), its segregant strain N2 (*red*), used in the evolution experiment, and the evolved strains NV3 (*green*), NV6 (*orange*), and NV15 (*blue*).

The 35 selected colonies from the evolution experiment were streaked in plates, and individual clones were selected and subjected to a phenotypic characterization. It consisted in microfermentation experiments in which 15 mL of the same synthetic must were dispensed in 50 mL conical tubes and capped with fermentation locks filled with mineral oil. Fermentation kinetics were monitored by daily recording weight loss. From the 35 selected colonies, three strains were selected according to two parameters. First, to show higher growth compared to the parental strain N2 but lower than the original strain EC1118. Second, to show differences between them, covering the fermentation performance spectrum from N2 to EC1118. From low to higher fermentation capacity, the selected strains were NV6, NV15, and NV3 (**Figure 1**).

### Growth Kinetics

Turbidity assays were also used to generate growth kinetic profiles in order to phenotypically characterize different strains. Growth kinetic profiles were run in triplicates, from independent inocula, starting from an initial OD_600_ of 0.2. Yeast cells were grown in 16 mm ID tubes filled with 7 mL of the appropriate media (synthetic must medium or YPD as described above), and turbidity was measured every hour, from 14 to 24 h after inoculation, by using a 2100N Turbidimeter (HASH, Loveland, CO, United States).

### Sequencing and Bioinformatic Analysis

Whole-genome sequencing and bioinformatic analysis were performed at the BGI (Beijing Genomics Institute, Shenzhen, Guangdong, China). Pair-end 90-bp reads were collected on an Illumina Genome Analyzer II platform giving 7.8 million reads per strain and covering around 95% of the genome of EC1118 that was used for mapping purposes. All sequencing data have been deposited in NCBI under the SRA accession number: SRP136088; and BioProject ID: PRJNA439262. After reads were processed, SOAPaligner (version 2.21) (Li et al., 2009) was used to align reads to reference sequence (*S. cerevisiae* strain EC1118) and SNPs and InDels calling and annotation. A common set of filters including quality score, read depth (minimum and maximum), and frequency of base calls at each position was taken into account for SNP calling. Thresholds were established to ≥5× read depth per position with ≥80% concordant SNP calls in the evolved and parental genome with a maximum of 200× read depth.

Coverage across reference tool in Qualimap v.0.8 (García-Alcalde et al., 2012) was use to analyse the possible aneuploidies.

### Removing of Chromosome XII Aneuploidy From the Parental Strain

The haploid parental strain N2 showed an aneuploidy in chromosome XII. In order to analyse the effect this aneuploidy had on the strain, the CCNV of the strain was altered by treating the strain with the antifungal agent methyl l-(butylcarbamoyl)-2-benzimidazolecarbamate, also known as benomyl. This drug is a well-known aneuploidy-inducing agent that destabilizes microtubules during mitosis and meiosis, affecting among other processes chromosome segregation, and has been extensively used for the study of aneuploidies in yeast (Bilinski et al., 1984; Taylor-Mayer et al., 1988). Parental strain N2 was treated with Benlate^®^ (50% concentration of benomyl), provided by Dupont (E.I. Du Pont de Nemours & Co., Wilmington, DE, United States). Yeast colonies were grown overnight at 28°C in the presence of four different drug concentrations with benomyl-free cultures of N2 as a control based on the protocol from Blasco et al. (2008). In short, a pre-inoculum of 48 h of N2 was used to inoculate at a final OD_600_ nm of 0.2 two replicates for each concentration of benomyl (5, 10, 20, and 40 μg/mL). Strains were grown overnight at 30°C in an orbital shaker at 150 rpm. Samples from the different concentrations were spread out onto YPD plates at appropriate dilution to allow single colonies to grow. Randomly selected colonies from each concentration were phenotypically characterized using turbidity assays.

The strains selected were isolated from the benomyl 40 μg/L treatment and were named N2b1 and N2b2.

### Sporulation and Spore Dissection

Cells were incubated (25°C, 150 rpm) in sporulation medium (1% KAc) after an overnight in pre-sporulation medium (YPG), in flasks allowing aeration at 250 rpm. When ascospores were visible by microscope at enough density, tetrads were dissected as follows: Ascus were digested with a zymolyase solution (2.5 mg/mL of zymolyase, 1 M sorbitol). A total of 500 μL of the solution with the sporulated cells was spun down and the cell pellets were resuspended in 500 μL of zymolyase solution (0.5 mg/mL) and incubated at room temperature for 10 min. Tetrad dissection was performed in a Singer micromanipulator (Sanger Scientific) and grown in YPD plates.

The strains generated by sporulation and spore dissection coming from the NV3 tetrad ascospore are named SpNV3.1, SpNV3.2, SpNV3.3, and SpNV3.4. Those dissected from the NV15 triad ascospore (no tetrads were found in NV15 sporulation assay) were named SpNV15.1, SpNV15.2, and SpNV15.3.

### Generation of Petite Strains

An overnight YPD liquid pre-culture is transferred to YPD liquid with 25 μg/mL of ethidium bromide and incubated 48 h at 28°C at 150 rpm. After this, a second step with the same incubation conditions is done. A 1 mL aliquot is washed twice and resuspended in the same medium before serial dilutions are made. Dilutions 10^-4^ and 10^-5^ are plated in YPD and incubated at 28°C. Small colonies are then transferred to new YPD and YPG plates to confirm the petite strains by their truncated ability to grow under glycerol as sole carbon source.

### Strain Identification by Delta Elements Fingerprinting and *MAT* Locus

The DNA extraction for PCRs was performed as described by Lõoke et al. (2011). During the evolution, strain identification was performed by using delta elements fingerprinting (Legras and Karst, 2003) in order to confirm that the culture remained without contamination (data not shown). Mating type PCR was run following the instructions of Huxley et al. (1990). The ancestral haploid strain N2 (*MAT*α) and the diploid strain EC1118 (*MAT*a/α) were used as controls. Evolution experiments time points were: 0, 50, 100, 150, 180, 200, 250, 263, and 273 cell generations.

### Quantitative PCR

Quantitative PCR (qPCR) was performed in order to study gain and loss of aneuploidies. The DNeasy plant kit (Qiagen, Valencia, CA, United States), was used for genomic DNA extraction. Some of the primers are based on the work from Pavelka et al. (2010) while the other primers were designed using Primer Quest following the recommendations in that work to target each chromosomal arm (**Table 1**). To study primers specificity and sensitivity, quality control was performed. All primer sets were evaluated using a four-point standard curve with eight replicates in order to get the amplification efficiency and dynamic range.

**Table 1:** Primers used for qPCR to study chromosome copy number variation.

Chromosomal arm (gene)		Sequence	Source
XII Right (YLR060)	F	CGGTATGGAAATGGACGAAGA	This work


	R	GGCTCCTCCCCGGTTTT	
X Left	F	ATTTACCGGTTAGTGTCAGCGCCA	Pavelka et al., 2010


	R	CGACAGAGTAGTTTATGCCGAGGGTT	
IV Left	F	AGCCCTAGTTGCAGATCATCGTGT	Pavelka et al., 2010


	R	AGAATATACGGCAACAGTGCCCGA	
IV Right	F	GGCCAACAAATCTTGTACCTCGCT	Pavelka et al., 2010


	R	GTTACCGAAGAAGGCCACCAATCT	
XVI Right (YPR002)	F	TCATGCCCCAAATACTGGATCT	This work


	R	AGCCCTCGAAACTGCATCTC	


*Between brackets: when amplified region targets an ORF.*

All qPCR experiments were conducted on the ABI Prism 7500 sequence detection system (Applied Biosystems group) using SYBR green chemistry. qPCR reactions were set upon five replicates, each with a reaction volume of 20 μL in a 96-well plate.

The Master mix for each sample was made using 10 μL of Power SYBR Green Mix (Applied Biosystems) 0.25 μM of each primer, 4 μL H_2_O and 5 μL of DNA.

The condition for the thermal cycle was as follows: 1 cycle of pre-incubation a 50°C for 2 min, followed by one cycle of 95°C for 10 min, and 40 cycles of amplification (95°C for 15 s and 60°C for 1 min).

Chromosome copy numbers were determined using the ΔΔCt method. The threshold cycle (Ct) value of each tested strain sample was first normalized against the control region, chromosome X, since it did not show any aneuploidy during the evolution experiment and was present in a single copy at N2 and at the evolved strain NV3. Then, the ΔCt value was calculated between the tested strain sample and relative to one of the benomyl treated strains (previously predicted to be haploid by FACs) to confirm the single copy of the chromosomes IV, XII, and XVI.

### Ploidy Estimations by Flow Cytometry

The DNA content of the strains studied in this work was assessed by flow cytometry by using the SYTOX Green dye method described in Haase and Reed (2002) in a Beckman Coulter FC 500 (Beckman Coulter Inc., Brea, CA, United States). Ploidy levels were scored on the basis of the fluorescence intensity compared with the haploid (S288c) and diploid (EC1118) *S. cerevisiae* laboratory and industrial reference strains (Mortimer and Johnston, 1986; Novo et al., 2009).

## Results

### Evolution Experiment of an Industrial Segregant Strain With Low Fermentation Efficiency

The aim of this work was to elucidate the adaptive mechanisms undergone by a wine yeast strain under industrial conditions. Specifically, by mimicking the first stage of a wine fermentation process, where cells grow exponentially. Since commercial wine strains are well adapted to grape fermentation conditions, we decided to start with a strain susceptible to improvement. For these reasons, the wine yeast strain EC1118 was chosen to generate derivative haploid segregant strains showing a lower efficiency compared to the parental strain.

The industrial strain EC1118 was sporulated and the haploid segregant strains were grown in synthetic must media. From the different segregants tested, the strain N2 was selected due to its impaired growth compared to the parental strain (**Supplementary Figure S1**).

The selected strain was grown in a bioreactor for approximately 270 generations in synthetic grape media. Continuous cultures were designed to mimic the early stages of grape must fermentation, as in our previous work with the laboratory strain BY4741 (Mangado et al., 2015).

Individual evolved yeast clones were selected at the end of the experiment and their fermentation performance tested in synthetic grape must compared to the initial N2 segregant strain and the EC1118 parental strain. In all cases, evolved strains showed improved fermentation performance compared to N2 but none of the individual clones exceeded the original EC1118 parental strain. About half of the tested clones showed a petite phenotype that was confirmed by their inability to grow in glycerol as the only carbon source. These clones were discarded for further analysis. However, in order to rule out respiratory deficiency as the main driver in adaptation to early stages of synthetic must fermentation, petite strains were generated from the segregant strain N2 (by using the ethidium bromide technique). Nevertheless, the N2 petite strains showed lower fermentation performance than N2 (**Supplementary Figure S2**).

From the evolved clones, three strains showing phenotypic differences in synthetic must were selected for genome sequencing). Strains were selected according to two parameters, first, to show higher growth compared to the parental strain N2, and second, to show differences between them, covering the fermentation performance spectrum from N2 to EC1118. From lower to higher fermentation capacity, the selected strains were NV6, NV15, and NV3 (**Figure 1**).

### Adaptation to Growth Conditions Occurs at Different Genomic Levels, Promoting Diploidization, Aneuploidies, *MAT* Locus Switching and SNPs

**FIGURE 2:**
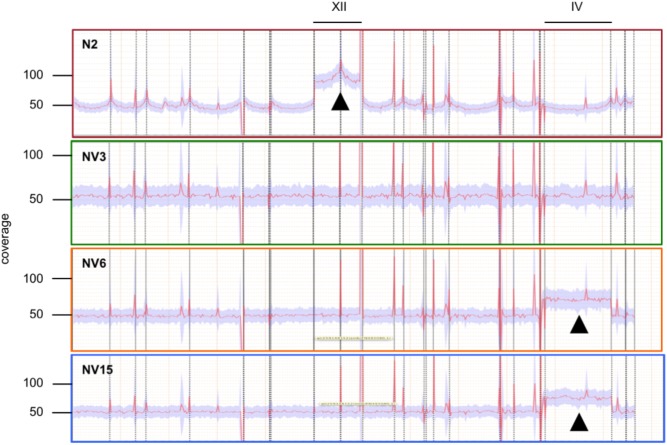
Coverage across the reference analysis of strain N2 (*red*), and the evolved strains NV3 (*green*), NV6 (*orange*), and NV15 (*blue*). Deviations from the average coverage of the genome are indicated by *black triangles*.

Average coverage from the genome sequencing results indicated that the segregant N2 strain had an aneuploidy in chromosome XII that we had not detected previously (**Figure 2**). The three evolved strains had lost this aneuploidy during the adaptation to first stage fermentation in synthetic must but instead, strains NV6 and NV15 gained an aneuploidy in chromosome IV. The aneuploidy in chromosome XII of the N2 strain had, on average, double the coverage compared to the rest of the genome, the same as in a haploid genome that had an extra copy of the chromosome XII. This was not the case for the aneuploidy of NV6 and NV15. These aneuploidies in chromosome IV have only around 50% more reads mapped on average compared to the rest of the genomes. These results can be explained if these strains have at least a 2n genome with an extra copy of chromosome IV.

**FIGURE 3:**
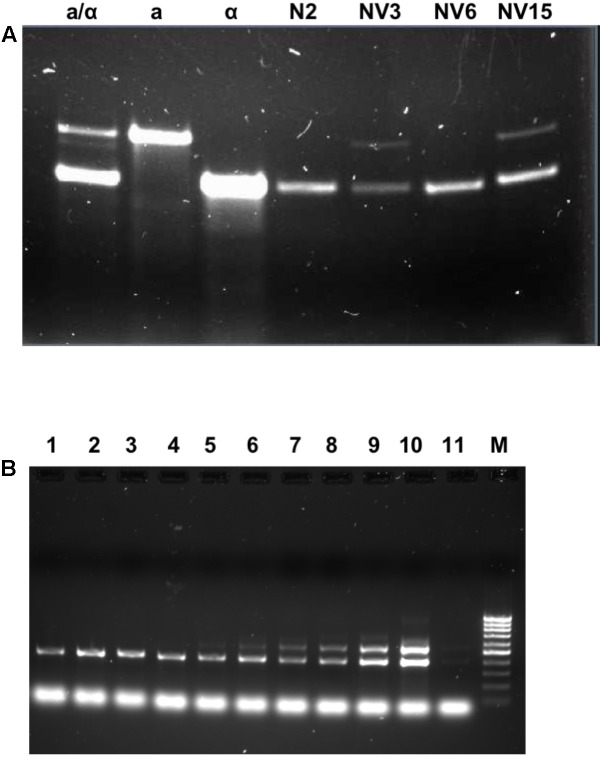
Mating type in the segregant and evolved strains. **(A)** The mating type by PCR amplification of the *MAT* locus for the different strains. First three lines are controls for *MAT*a/α (BY4743), *MAT*a (BY4741), and *MAT*α (BY4742), then, the segregant strain N2 *MAT*α *ho*, and the evolved strains NV3 *MAT*a/α, NV6 *MAT*α, and NV15 *MAT*a/α. **(B)** The mating type by PCR amplification of the *MAT* locus for different time points during the evolution experiment. From *line 1* to *line 9*, the evolving pool at generations 0, 50, 100, 150, 180, 200, 250, 263, and 273; *line 10*: positive control EC1118; *line 11*: positive control EC1118 (1/100 dilution); and *line M*: 100 bp marker.

To clarify these results, the strains were subjected to a ploidy analysis. The results can be seen in **Supplementary Figure S3**. The three evolved strains have undergone a duplication of the whole genome. Instead of a 1n genome like the N2 segregant strain from which they were derived, they have a 2n genome. Moreover, despite the *ho* genotype, two of the three evolved strains have switched the *MAT* locus. Although this switch happens for unknown reasons, it has been described to occur spontaneously once per million cells (Ira, 1983). The evolved strain NV6 is still *MAT*α like its ancestor N2, while NV3 and NV15 after the evolution experiment are *MAT*a/α (**Figures 3A,B**). A possible mutation of the gene *HO* from the inactive form (*ho*) to the active form (*HO*) was ruled out by sequence alignment. No mutations were detected in the evolved compared to the parental *ho* strain (**Supplementary Figure S4**).

Genome sequencing also revealed SNPs and InDels present in the evolved strains, most of them in heterozygosis and shared among the three strains (**Supplementary Figure S5**). Those in homozygosis and therefore that could have a greater impact than those in heterozygosis were, a common SNP to all three evolved strains displayed in the *UTP14* gene, a gene related with ribosomal RNA processing. Then, in the strain NV3, one unique SNP, in the mitochondrial gene *COX1* and, also in this strain, there was a unique deletion in gene *PET127*, involved in processing mitochondrial RNA. Finally, in strain NV6 another small mitochondrial deletion in the gene *COX3*. All these homozygous changes were pointing to respiration as a target of the evolution experiment. To test if there was a significant difference in the respiration performance of the three evolved strains, we measured the ratios between respiration and fermentation based on the uptake and production of O_2_ and CO_2_. The results of this experiment did not show any significant difference in the Respiration Quotient of the evolved strains (Data not shown).

### Diploidization Effect on Growth Performance Depends on the Media Conditions

**FIGURE 4:**
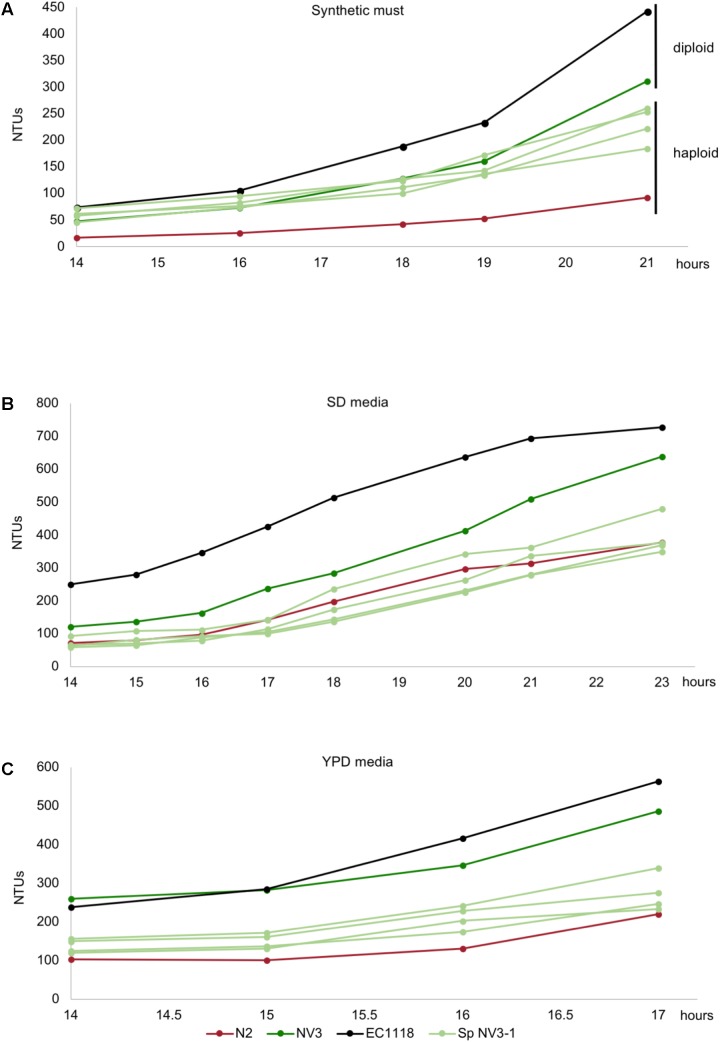
Growth kinetics in synthetic grape must **(A)**, SD media **(B)**, and YPD media **(C)** of the original industrial strain EC1118 (*black*), its segregant strain N2 (*red*), the evolved strain NV3 (*green*), and four spore derivatives from the same ascus of NV3 (*light green*).

In order to evaluate the effect of diploidization in the adaptation of the evolved strains to a first stage fermentation in synthetic must, these strains were subjected to sporulation. Only strains NV3 and NV15 were able to sporulate, with NV15 being much slower compared to NV3. Spore viability in NV3 was around 90% (from 14 dissected asci) with many asci composed of four spores, while the viability in NV15 was only 60% (from 14 dissected asci), only achieving up to three spores per ascus. Spore clones obtained from the four-spored asci of NV3 were supposed to be haploid because they inherited inactive *ho* alleles and NV3 had no aneuploidies. In the case of NV15, with an aneuploidy in chromosome IV, the lack of four-spored asci, indicates aberrant meiosis and therefore, despite the FACS results showing a haploid genome (**Supplementary Figure S3**), it is possible that spores from NV15 could present some aneuploidies. Four spores belonging to the same ascus from NV3 and three spores belonging to the same ascus from NV15 were selected and cultured in different media. As shown in **Figure 4A**, growing in synthetic must, none of the haploid strains derived from the evolved diploid strain NV3 reaches the performance of the parental strain. At this time point (21 h), haploid strains show a percentage of growth compared to EC1118 ranging from 41 to 58%, whereas the diploid NV3 strain reaches 70%. Still, the haploid strains show double growth compared to the original parental strain N2 (20% of EC1118 growth). Thus, haploid strains derived from the evolved NV3 strain are not as efficient in growth as the diploid strain but maintain most of the adaptation gained during the evolution experiment by NV3. It must be taken into account that the heterozygosity of NV3 could in part explain the phenotype and it is therefore difficult to conclude that the observed differences are exclusively due to the ploidy effect. Interestingly, the same strains were grown in different media (SD and YPD) and the advantage observed in synthetic must media was lost there (**Figures 4B,C**). The haploid strains behave similarly to the original parental strain N2, meaning that, in these media, ploidy has an effect on growth. Therefore, the effect of ploidy on the strains depends on the growth conditions, being weaker in the original selection conditions.

**FIGURE 5:**
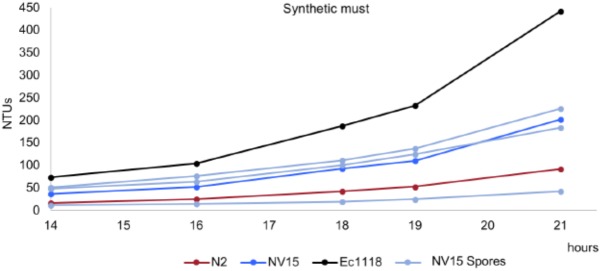
Growth kinetics in synthetic grape must of the original industrial strain EC1118 (*black*), its segregant strain N2 (*red*), the evolved strain NV15 (*blue*), and three spore derivatives from the same ascus of NV15 (*light blue*).

In the case of NV15, the aneuploidy in chromosome IV must be interfering in the meiosis process and preventing the strain from sporulating correctly. Even so, the results for the three spores from each ascus were always similar, with one of the spores showing a very low growth performance, lower than N2 (**Figure 5**), and the other two with similar values compared to the diploid strain, NV15. Therefore, in this case, the ploidy level seems to have no effect. Although we can not rule out that the result is influenced by possible aneuploidies present in the spore clones due to an aberrant meiosis.

### Aneuploidy of Chromosome XII Has a Detrimental Role on Growth Efficiency

**FIGURE 6:**
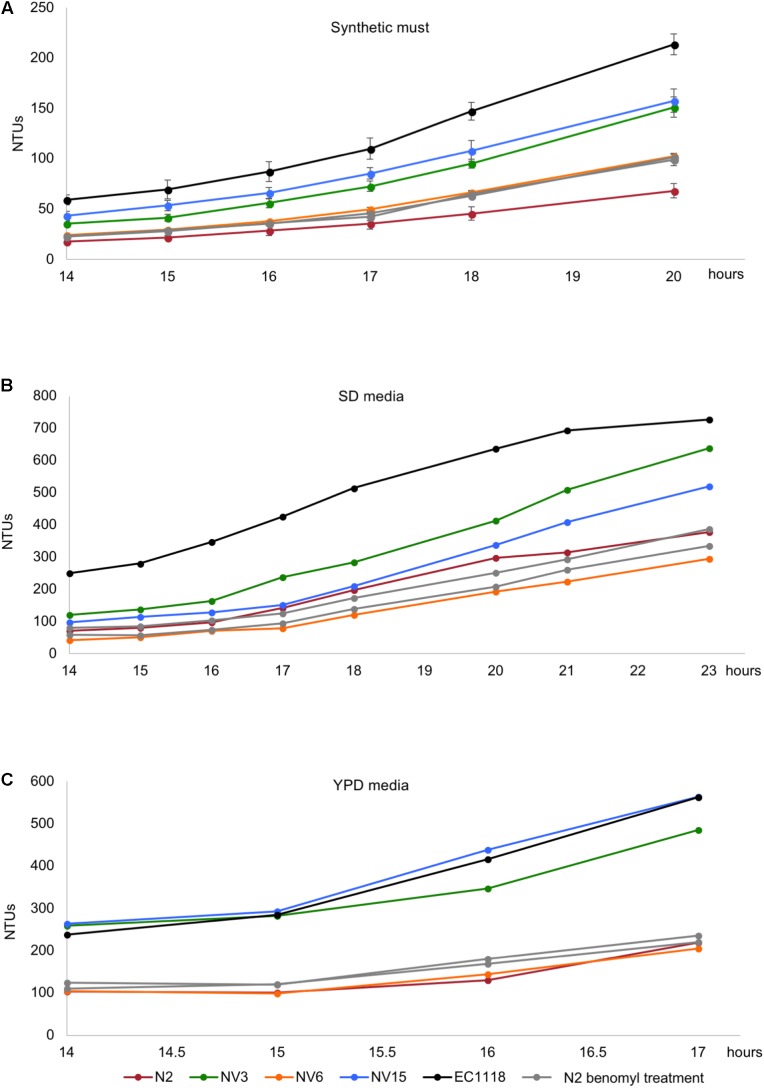
Growth kinetics in synthetic grape must **(A)**, SD media **(B)**, and YPD media **(C)** of the original industrial strain EC1118 (*black*), its segregant strain N2 (*red*), the evolved strains NV3 (*green*), NV6 (*orange*), NV15 (*blue*), and two strains isolated from the benomyl 40 μg/L treatment (*light gray*).

After diploidization, aneuploidies are the next major change in terms of genomic rearrangements. We wanted to evaluate the effect that the different gain and losses of chromosomes had on the adaptation to our cultivation conditions. We first addressed the effect of the chromosome XII aneuploidy present in the founder strain N2. For this purpose, the N2 strain was subjected to four different concentrations of benomyl, a well-known aneuploidy-inducing agent, which affects among other processes, chromosome segregation and has been extensively used for the study of aneuploidies in yeast (Taylor-Mayer et al., 1988). Random colonies were selected and screened in synthetic must, looking for strains that had an improved phenotype, based on the initial hypothesis that aneuploidy on chromosome XII in our founder strain was an important factor in its lower growth efficiency compared to EC1118. Different strains with an improved phenotype were selected. Then, a ploidy analysis was run detecting both, haploid and diploid strains. Haploid strains were selected (**Supplementary Figure S3**) for qPCR analysis, to verify that the extra chromosome XII was lost (**Supplementary Figure S6**). N2b1 and N2b2 were confirmed to be haploid derivates from N2 without the aneuploidy in chromosome XII. The growth of these strains in different media was analyzed. As can be seen in **Figure 6**, the two strains selected from the benomyl experiment growing in synthetic must have an enhanced phenotype compared to N2. The improvement in growth due to the loss of the aneuploidy is not observed when the strains grow in SD or YPD, with their growth very similar to N2 or NV6 in both cases (**Figures 6B,C**). These results clearly indicate the detrimental role of chromosome XII in the growth efficiency of N2 in synthetic must. Despite the growth improvement, which makes N2b1 and N2b2 similar in terms of growth efficiency to the evolved strain NV6, they are still far from strains NV3 or NV15.

### Possible Evolutionary Events in Perspective

**FIGURE 7:**
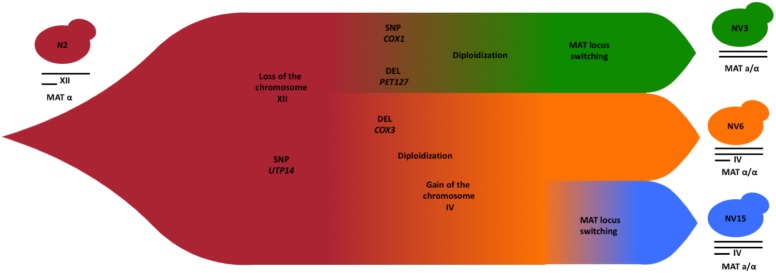
Schematic diagram showing the chronological order of the evolutionary events from the segregant strain N2 to the evolved strains NV3 (*green*), NV6 (*orange*), and NV15 (*blue*).

Based on the results reported above, we can hypothesize the approximate chronological order of the evolutionary events that have occurred to give rise to the three strains after evolution (**Figure 7**). The extra copy of chromosome XII in the parental haploid strain N2, could have been lost early in the evolution experiment, since, it is detrimental in the growing conditions of the experiment and does not appear in the evolved strains. The analysis of the heterozygosity of chromosome XII in the different strains supports the loss of this chromosome before the duplication event, otherwise the level of heterozygosity of chromosome XII would have been higher compared with the rest of the genome, more similar to the heterozygosity that N2 shows for this chromosome (**Supplementary Figure S7**). The common SNP shared by the three strains in homozygosis, the gene *UTP14* found at chromosome XIII, could appear before the genome duplication events, when the population had still a single copy of this chromosome and, before the population diverged in the at least three studied strains. During the genome duplication, this SNPs would have been fixed in homozygosis and remained in the diverged strains. After these events, the simplest hypothesis to explain the next evolutionary steps are deletion in *PET127*, located in chromosome XV and the mitochondrial SNP in *COX1*. These changes will start to differentiate strain NV3, while a deletion in *COX3* will do the same for a subpopulation that eventually will become NV6 and NV15. In both cases, a diploidization event, independently in both subpopulations fixed these SNPs in homozygosis. Before or after this diploidization, the NV3-6 subpopulation would have gained an extra copy of chromosome IV, common in both strains. Finally, a *MAT* locus switching in NV3 and NV15 but not in NV6 will ultimately define the evolved strains that were finally selected. According to the sequencing data, both parental and evolved strains are *ho*, so all the diploidization, and notably mating type switching events, must have taken place in the absence of *ho* reversion.

## Discussion

### Adaptation to Growth Conditions Occurs at Three Different Levels

This work intends to provide knowledge about the adaptation mechanisms that occur in yeast cells during growth in synthetic grape media, specifically at the start of the fermentation process when sugars and nitrogen are widely available and cells are at their maximum duplication rate. Evolution experiments using continuous cultures were conducted in bioreactors for approximately 270 generations. In a previous work with the lab strain BY4741 (Mangado et al., 2015), the main adaptation observed was related to an intrinsic characteristic of the strain background. In this second approach, a derivative of an industrial strain was used. We selected a segregant strain derived from a well-known industrial strain that showed lower fermentation capacity compared to the parent strain. This lower fermentation capacity provides the possibility of an improvement margin for this segregant during the continuous culture evolution experiment.

The respiratory deficient or petite phenotype under fermentation conditions is a known problem Castrejón et al. (2002) that renders it impossible for yeast cells to be reused once fermentation is finished, among other things due to the large increase in petite strains (Sanchez et al., 2012). Although the specific conditions are not known, they have been related to the stress conditions derived from the fermentation process. The growth under high acetaldehyde and ethanol content will expose the mitochondria to high levels of reactive oxygen species (ROS), eventually leading to DNA damage. The higher rate of these mutations compared to the nuclear DNA rate is explained by physical proximity to the point where ROS are generated (Castrejón et al., 2002; Smart, 2007; Gibson et al., 2008). Our experimental conditions reproduce the first stage fermentation process, meaning that there are no high levels of ethanol or acetaldehyde, but rather there is a high osmotic stress with a sugar content near 200 g/L. Until now, the appearance of this phenotype during wine fermentation has been related to stress resulting from the accumulation of secondary metabolites derived from the yeast fermentation metabolism, with this the first work that also identifies it under conditions of first phase fermentation in ALE. The high observed frequency of petite strains after 270 generations indicated that this phenotype could confer an advantage in our experimental conditions. The generation of petite strains from the segregant strains did not confirm this advantage in single batch experiments and therefore opens the possibility that the high number of respiratory deficient strains is only the result of the accumulation of mutations in the mitochondrial genome derived from the growth under stress conditions. This could also explain the mitochondrial SNPs observed in *COX1* and *COX3* in the evolved strains selected. The other SNP found in *PET127* is also related with the petite phenotype and its deletion genetic background of the lab strain BY4741 leads to the appearance of this kind of mutants. In our case the SNP does not produces this phenotype, meaning that no loss of function (LOF) is detected or that it has no effect under this genetic background. This gene codifies for a protein with a role in the processing of mitochondrial RNAs. The observed change could be related to an adaptation to cope with hypothetical mitochondrial DNA stress by changes in the processing of the mitochondrial RNAs. Although more SNPs were identified, they were mostly found in heterozygosis, making it difficult to assess their effect in this context.

Genome duplication as an adaptation phenomenon has been observed before (Oud et al., 2013), as other works have also mentioned, diploidization could be seen as a faster way to adapt to a new environment, providing the opportunity for heterozygous gene changes to occur (Turunen et al., 2009). In this work, the genome duplication is an advantage over the haploid strains, at least in the case of the evolved strain NV3, the only one that sporulated efficiently. Aneuploidies affect sporulation efficiency (Sunshine et al., 2016), as in the case of the NV15 strain, making difficult to evaluate the effect of diploidization. Although the effect of SNPs in heterozygosis in NV3 might mask the effect. Curiously, diploidization gave a much clearer advantage when the strain grew in environments it had not been adapted to. In the other media assessed, the haploid strains have the same efficiency as the parental N2 strain, while the evolved diploid strain grew better. It could be that the SNPs in heterozygosis emerged in response to the adaptation to the conditions of the experiment are detrimental in other culture media, thus conferring an advantage to the diploid strain, since in this case it contains only one of the two alleles that have been modified.

The three evolved strains underwent a diploidization phenomenon although only one of them retained the original mating type. The other two strains experienced independent events of mating type switching. Our selected parental strain was *ho MAT*α strain and was expected to be a stable haploid strain. The heterothallism of the strain was confirmed by sequencing of the *MAT* locus. It is worth mentioning that the original EC1118 strain is heterozygous for the *HO* locus. Cells with the recessive *ho* allele switch mating type only at low frequency, approximately one per million cells (Ira, 1983). It could be that the diploid genotype *MAT*a/α confers a selective advantage under our experimental conditions. Indeed, the two diploid strains showing a *MAT*a/α genotype show higher fermentation performances compared to the *MAT*α/α strain. In terms of sexual phenotype, isogenic diploid strains, a/a or α/α will behave like haploid strains (Paquin and Adams, 1982). Our data would concur with this hypothesis since the two closest evolved strains differ in the *MAT* locus, having a higher efficiency in growth the *MAT*a/α compared to the *MAT*α/α strain. As mentioned before, the SNPs in heterozygosis of these two strains do not allow us to confirm this hypothesis.

Industrial winemaking strains are commonly found to be diploid and to possess aneuploidies (Sipiczki, 2011). Together with genome duplication events, chromosomal rearrangements have also been identified as a major source of variation in adapting to new environments conditions (Kohn, 2005; Chen et al., 2012; Voordeckers and Verstrepen, 2015). In the work of Chang et al. (2013) chromosomal rearrangements contribute to extremely high copper tolerance in a set of natural yeast strains. When conditions fluctuate and aneuploidies are no longer an advantage, they readily disappear from the population, conferring greater flexibility to the genomic rearrangements than point mutations. Sipiczki (2011) refers to this unique plasticity of the wine yeast genome as fast adaptive genome evolution (FAGE), proposing a model for the accumulation and loss of genomic rearrangements in the winemaking environment. In this work, and according to our results, the aneuploidy found in the parent strain is detrimental in the assayed conditions. Therefore, the genome duplication observed in our experimental set up, could be a way to mitigate the aneuploidy on chromosome XII of the parental strain. Here the aneuploidy was a burden for the growth in synthetic must and by eliminating it, the segregant strain improved its growth by matching it with one of the strains evolved over 270 generations, that is, with the strain that had improved its fermentation capacity the least. The burden of an aneuploidy turned out to be specific for growth in the media of evolution, synthetic must, since no effect was seen in the other media in which it was cultivated. Aneuploidy of chromosome IV could also be driven by the aneuploidy in chromosome XII, as it has been shown that CCNV induces genomic instability (Sheltzer et al., 2011).

This work shows the evolutive mechanism of three *S. cerevisiae* strains covering three different levels of adaptation, diploidization at genomic level, aneuploidies at the chromosome level, and SNPs at the gene level. We also show how aneuploidies can be beneficial or detrimental depending on the media conditions. Industrial yeast strains and in particular wine-yeast strains usually present CCNV (de Vries et al., 2017). These genetic particularities help them to cope with the industrial stresses that they are exposed to, and may be genomically stable only under industrial conditions, not conferring true advantages outside these particular environments. The aneuploidy in chromosome XII of the founder strain contributes to its minor growth efficiency compared to the original wine strain but only under synthetic must conditions. On the other hand, another aneuploidy, this time in chromosome IV, is part of the adaptation to the experimental conditions in two of the evolved strains. Also, diploidization seems to be a phenomenon to counteract the negative impact of aneuploidies while the *MAT* locus switch that seems to have happened in two independent instances could be a consequence of the genomic instability caused by the aneuploidies.

## Data Availability

The data set supporting the results of this article is available in the NCBI repository under Sequence Read Archive SRP136088 accession number BioProject ID: PRJNA439262. The data set supporting the results of this article is included in the article (and its Additional files).

## Author Contributions

JT, RG, PM, and AM conceived and designed the study. AM and JT performed the experiments and analyzed the data. JT, RG, and PM interpreted the results and wrote the manuscript. All authors discussed and approved the manuscript.

## Conflict of Interest Statement

The authors declare that the research was conducted in the absence of any commercial or financial relationships that could be construed as a potential conflict of interest.

## Supplementary Material

FIGURE S1 Fermentation kinetics in synthetic grape must of the original industrial strain EC1118 (*black*) and its segregant strain N2 (*red*).

FIGURE S2 Growth kinetics in synthetic grape must of the segregant strain N2 (*red*), and three strains derived from N2 after treatment with ethidium bromide, showing petite phenotype.

FIGURE S3 Ploidy estimations by flow cytometry of the different strains used in this work. *Upper panel*, cytometry plots of the segregant strain N2 (*red*), and the evolved strains NV3 (*green*), NV6 (*orange*), and NV15 (*blue*). *Lower panels*, raw and normalized by S288c FITC-A Geo Mean Average values.

FIGURE S4 Amino acid variants of gene YDL227C (HO). Amino acid sequence alignment of parental and evolved strains against the *ho* lab strain S288c. The conserved motifs of site specific endonuclease activity are shown.

FIGURE S5 SNPs and InDels of the evolved strains. *Upper panel*, SNPs in homozygosis and heterozygosis of the evolved strains. *Lower panel*, individual and shared SNPs in homozygosis for the evolved strains.

FIGURE S6 Relative quantification by qPCR of selected chromosomes of the segregant strain N2, the evolved strain NV3, and two strains from the benomyl 40 μg/L treatment. Chromosome IV relative quantification is in *light blue*, chromosome XII in *pink*, and chromosome XVI in *light orange*. Relative quantification against chromosome X and one of the benomyl treated samples.

FIGURE S7 Heterozygosity analysis. *Upper panel* shows the number of non-filtered SNPs in heterozygosis per kb for the segregant strain N2 (*red*), the evolved strains NV3 (*green*), NV6 (*orange*), and NV15 (*blue*). In the *lower panel* N2 has been removed for a better comparison between the evolved strains.
