# Supplementary figures and images for: Evolution of a Yeast With Industrial Background Under Winemaking Conditions Leads to Diploidization and Chromosomal Copy Number Variation
Source: Front Microbiol. 2018 Aug 3;9:1816. doi: 10.3389/fmicb.2018.01816 (PMC6088182; doi:10.3389/fmicb.2018.01816)

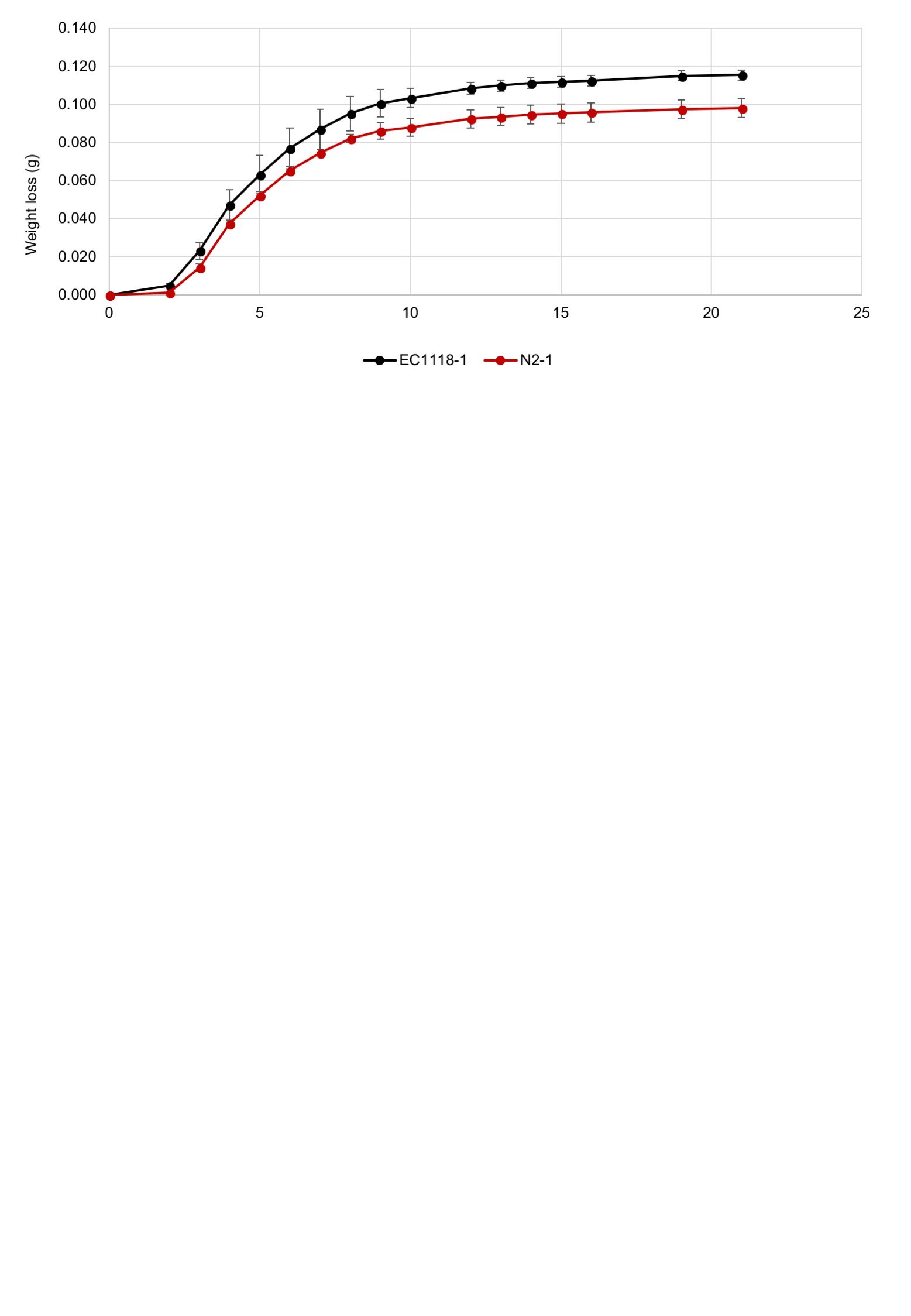

Supplement: FIGURE S1 — Fermentation kinetics in synthetic grape must of the original industrial strain EC1118 (black) and its segregant strain N2 (red). [file Image_1.TIFF]

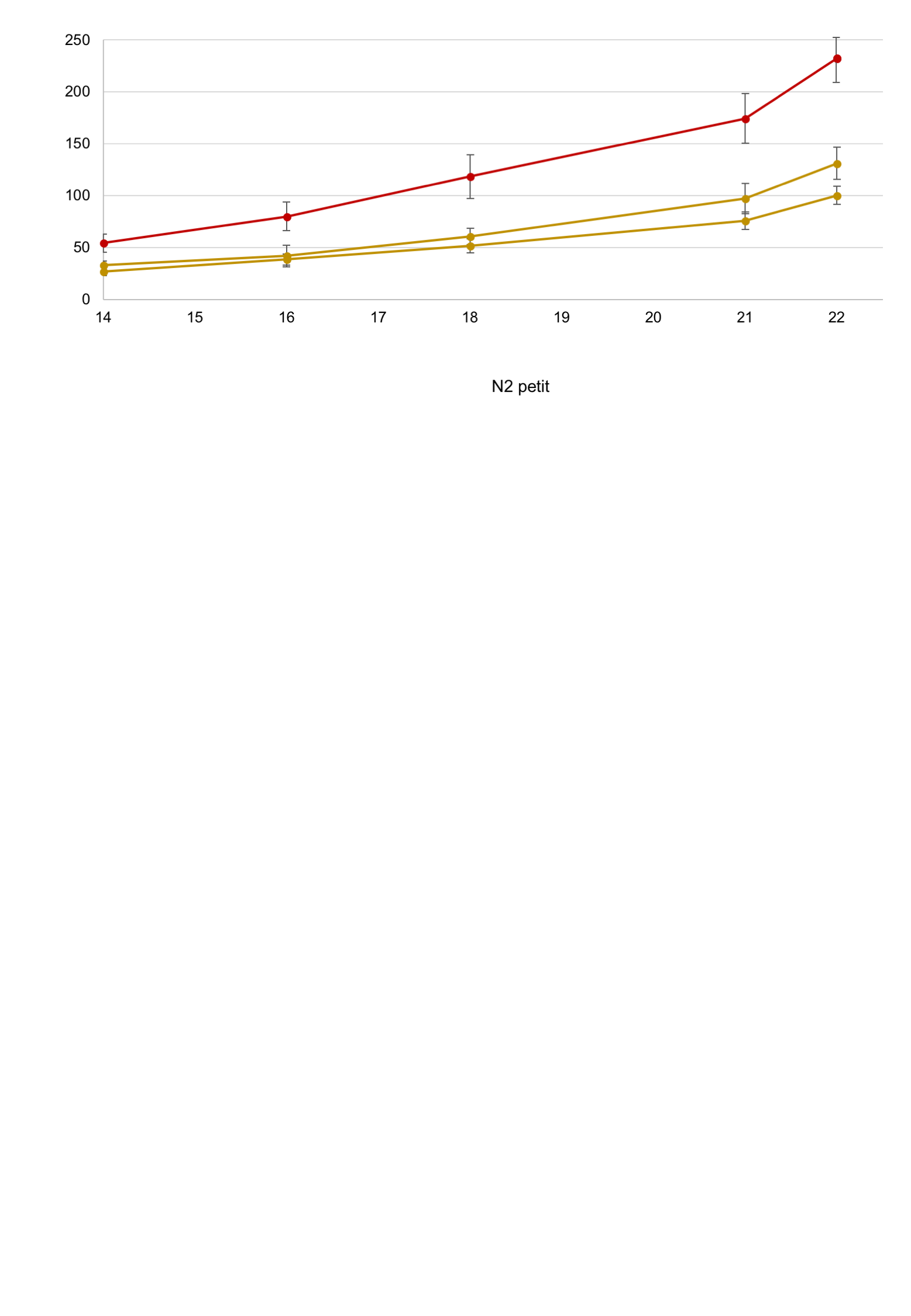

Supplement: FIGURE S2 — Growth kinetics in synthetic grape must of the segregant strain N2 (red), and three strains derived from N2 after treatment with ethidium bromide, showing petite phenotype. [file Image_2.TIFF]

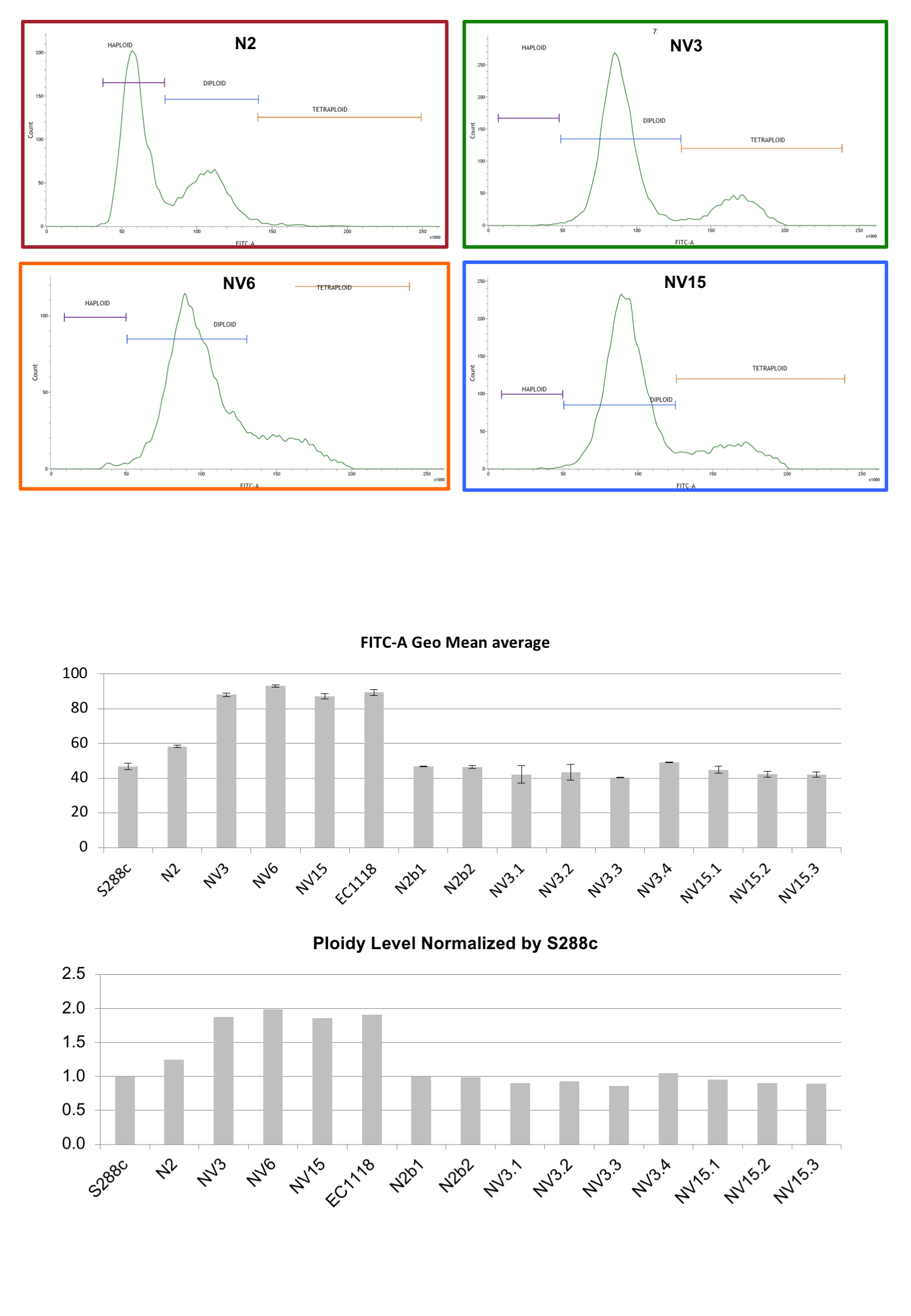

Supplement: FIGURE S3 — Ploidy estimations by flow cytometry of the different strains used in this work. Upper panel, cytometry plots of the segregant strain N2 (red), and the evolved strains NV3 (green), NV6 (orange), and NV15 (blue). Lower panels, raw and normalized by S288c FITC-A Geo Mean Average values. [file Image_3.TIFF]

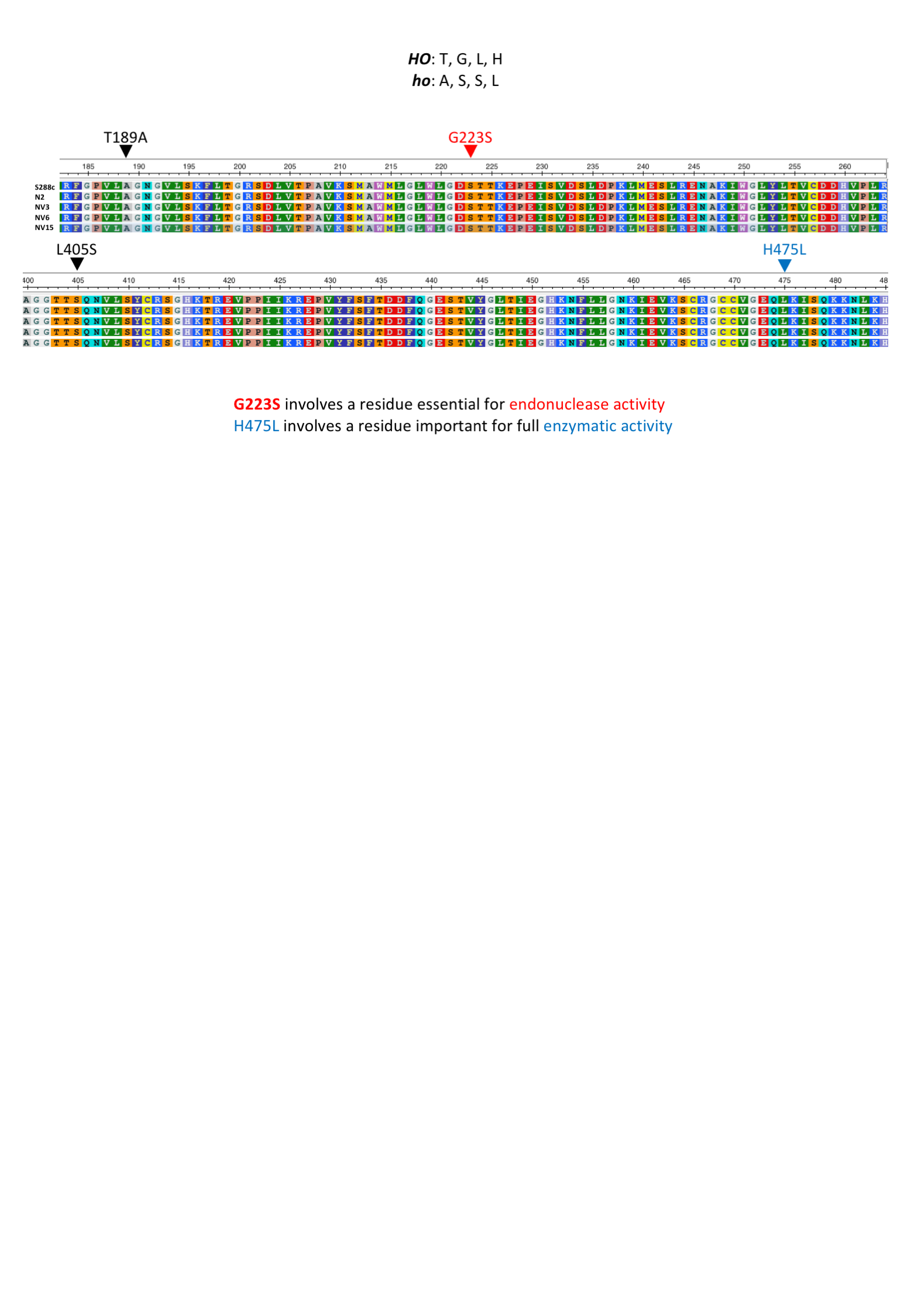

Supplement: FIGURE S4 — Amino acid variants of gene YDL227C (HO). Amino acid sequence alignment of parental and evolved strains against the ho lab strain S288c. The conserved motifs of site specific endonuclease activity are shown. [file Image_4.TIFF]

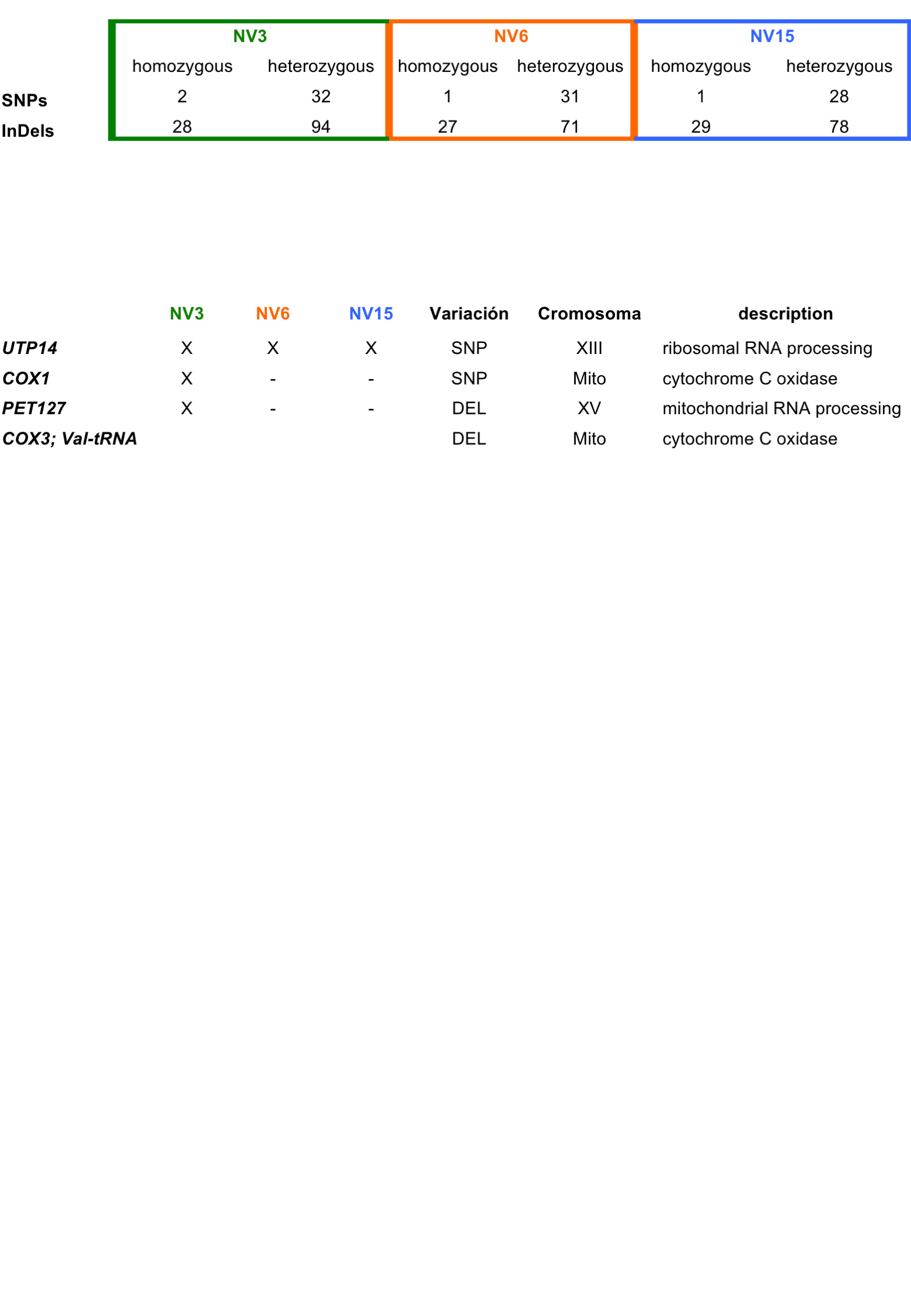

Supplement: FIGURE S5 — SNPs and InDels of the evolved strains. Upper panel, SNPs in homozygosis and heterozygosis of the evolved strains. Lower panel, individual and shared SNPs in homozygosis for the evolved strains. [file Image_5.TIFF]

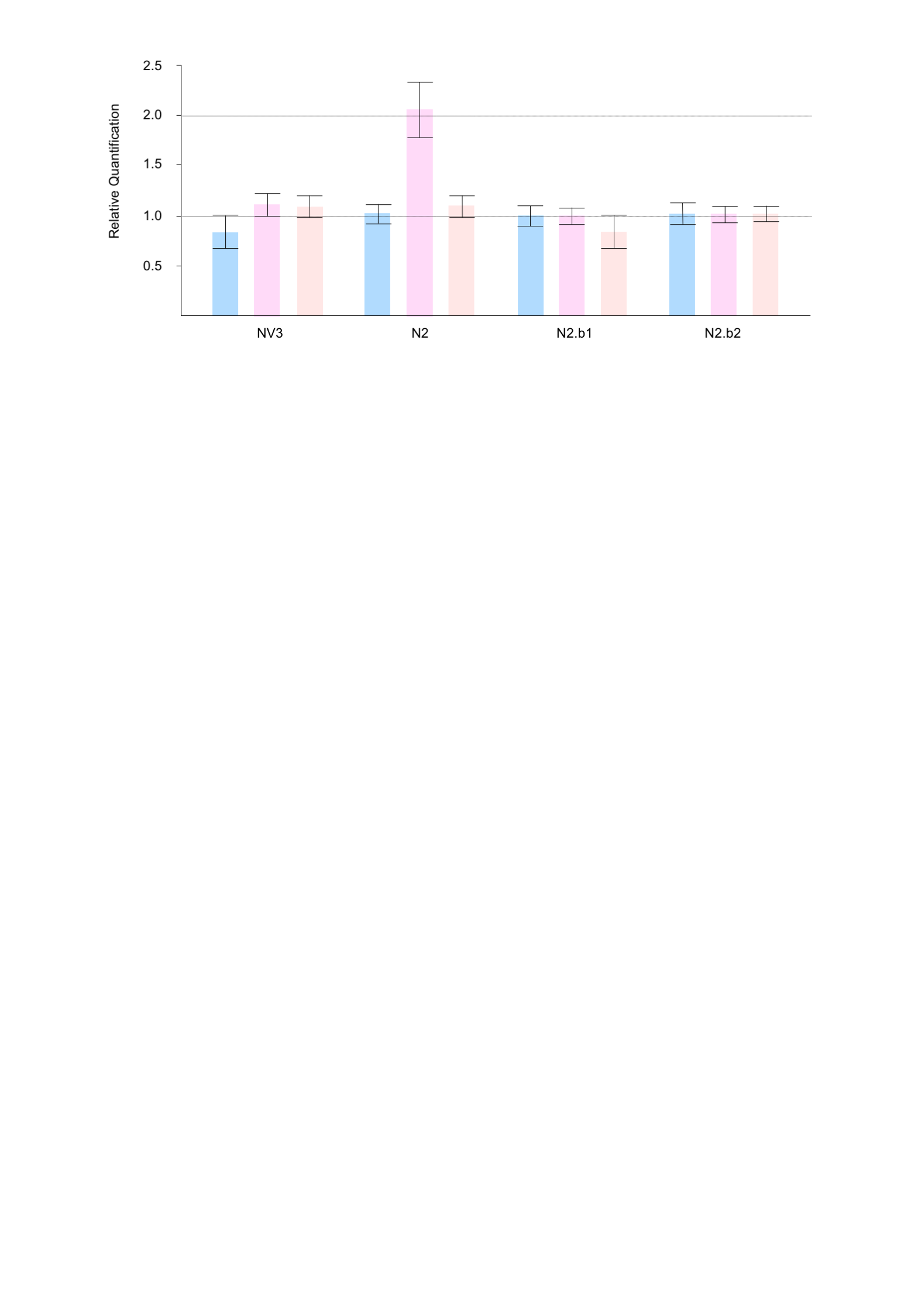

Supplement: FIGURE S6 — Relative quantification by qPCR of selected chromosomes of the segregant strain N2, the evolved strain NV3, and two strains from the benomyl 40 μg/L treatment. Chromosome IV relative quantification is in light blue, chromosome XII in pink, and chromosome XVI in light orange. Relative quantification against chromosome X and one of the benomyl treated samples. [file Image_6.TIFF]

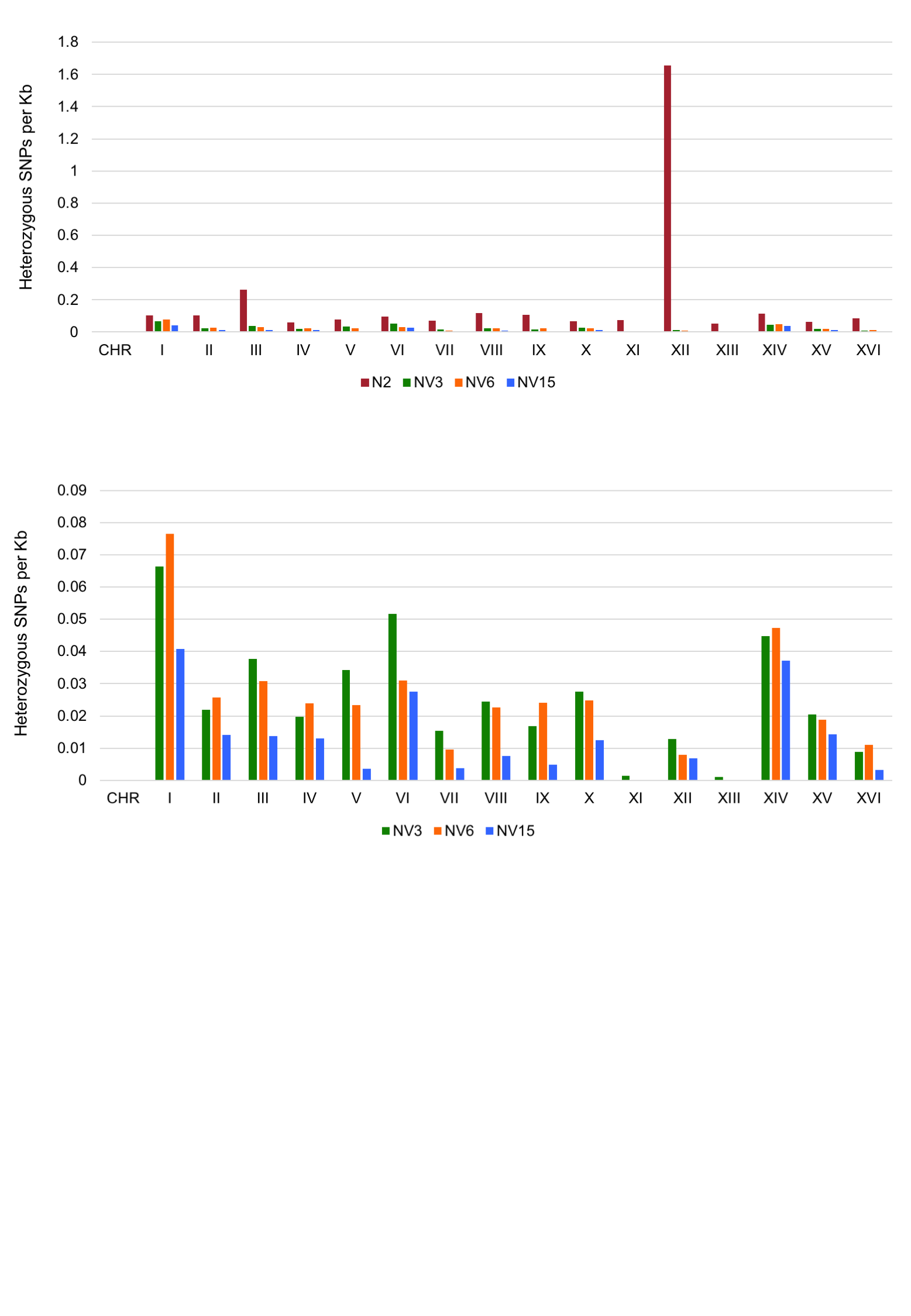

Supplement: FIGURE S7 — Heterozygosity analysis. Upper panel shows the number of non-filtered SNPs in heterozygosis per kb for the segregant strain N2 (red), the evolved strains NV3 (green), NV6 (orange), and NV15 (blue). In the lower panel N2 has been removed for a better comparison between the evolved strains. [file Image_7.TIFF]
